# Benralizumab in Patients with Severe Eosinophilic Asthma: A Multicentre Real-Life Experience

**DOI:** 10.3390/jcm12134362

**Published:** 2023-06-28

**Authors:** Giulia Scioscia, Pasquale Tondo, Santi Nolasco, Corrado Pelaia, Giovanna Elisiana Carpagnano, Maria Filomena Caiaffa, Giuseppe Valenti, Angelantonio Maglio, Francesco Papia, Massimo Triggiani, Nunzio Crimi, Girolamo Pelaia, Alessandro Vatrella, Maria Pia Foschino Barbaro, Claudia Crimi

**Affiliations:** 1 Department of Medical and Surgical Sciences, University of Foggia, 71122 Foggia, Italy; giulia.scioscia@unifg.it (G.S.); mariapia.foschino@unifg.it (M.P.F.B.); 2 Department of Clinical and Experimental Medicine, University of Catania, 95124 Catania, Italy; nolascos@hotmail.it (S.N.); crimi@unict.it (N.C.); dott.claudiacrimi@gmail.com (C.C.); 3 Department of Health Sciences, University “Magna Graecia” of Catanzaro, 88100 Catanzaro, Italy; pelaia.corrado@gmail.com (C.P.); pelaia@unicz.it (G.P.); 4 Department of Basic Medical Sciences, Neuroscience and Sense Organs, University “Aldo Moro” of Bari, 70121 Bari, Italy; elisiana.carpagnano@uniba.it; 5 Allergology and Clinical Immunology Unit, University of Foggia, 71122 Foggia, Italy; mariafilomena.caiaffa@unifg.it; 6 Allergology and Pulmonology Unit, Provincial Outpatient Center of Palermo, 90127 Palermo, Italy; vasvalenti@gmail.com (G.V.); dott.francesco.papia@gmail.com (F.P.); 7 Department of Medicine, Surgery and Dentistry, University of Salerno, 84084 Salerno, Italy; amaglio@unisa.it (A.M.); mtriggiani@unisa.it (M.T.); avatrella@unisa.it (A.V.)

**Keywords:** severe asthma, benralizumab, mepolizumab, switching, biologics

## Abstract

Background: Mepolizumab and benralizumab are monoclonal antibodies directed against anti-IL-5 and anti-IL5R, respectively, and their use reduces the exacerbation rate and maintains oral corticosteroid requirements in severe eosinophilic asthma. Previous studies have tested the therapeutic switch between two biologics with excellent results, further demonstrating the heterogeneity of asthmatic disease and the complexity of the therapeutic choice. It remains unclear if such patients may improve following a switch from mepolizumab to benralizumab. Aims: Within a multicentre real-life setting, we decided to evaluate the potential effectiveness of a therapeutic switch to benralizumab in patients with severe eosinophilic asthma initially treated with mepolizumab, who experienced sub-optimal responses. The secondary aim was to identify the clinical factors associated with a better response to benralizumab. Methods: We retrospectively assessed patients with severe eosinophilic asthma treated at six Italian specialist centres, who were switched from mepolizumab to benralizumab following a sub-optimal response, defined as a partial or total lack of clinical remission (i.e., frequent severe exacerbations and/or poorly controlled symptoms and/or higher OCS daily use in patients with a poor or moderate response in the global evaluation of treatment effectiveness scale), after at least 12 months of treatment. Results: Twenty-five patients were included in the analysis (mean age 56.76 ± 11.97 years, 65% female). At 6 months of treatment with benralizumab, the ACT score was significantly higher than the ACT score with mepolizumab (20.24 ± 3.38 vs. 16.77 ± 3.48, *p* < 0.0001); the mean number of daily SABA inhalations was significantly lower after 6 months and 12 months of treatment with benralizumab than that after treatment with mepolizumab; OCS intake and the prednisone median dosage at 6 months of treatment with benralizumab were significantly lower than those with mepolizumab. Benralizumab treatment resulted in a marked improvement in asthma control, suppressed blood eosinophil levels and reduction in the number of exacerbations in the subgroup of patients with severe eosinophilic asthma and nasal polyposis. Conclusions: Patients diagnosed with severe eosinophilic asthma who experience a partial response to mepolizumab could benefit from switching to benralizumab, and even more those who have nasal polyposis.

## 1. Introduction

Asthma is a chronic airway disease characterized by variable expiratory airflow limitation and bronchial hyperresponsiveness, sustained by chronic inflammation and remodelling of the airways [1,2]. Despite significant improvement in the diagnosis and management of this disorder, most asthmatic patients (5–10%) suffer from a severe form (SA), characterised by poor symptom control, despite medium or high dosages of inhalation treatment and courses of oral corticosteroids (OCSs) [3].

The discovery of the molecular mechanisms involved in the pathogenesis of asthma (endotypes) has led to the development of biologic therapies that have provided an effective therapeutic choice in SA patients, improving the quality of life by reducing exacerbations, improving lung function and reducing OCS intake, with a good safety profile [4,5]. 

Relevant eosinophilic airway inflammation is present in many patients with SA, regardless of their allergic condition [6]. Pharmacological research has focused on the development of targeted anti-eosinophil biological therapy strategies against interleukin-5 (IL-5) or its receptor, fulfilling a crucial unmet need for the treatment of CS-dependent severe eosinophilic asthmatic patients who are greatly affected by the side effects of OCS maintenance therapy. The first drug developed was mepolizumab, followed by reslizumab, both of which are anti-IL-5 monoclonal antibodies and, more recently, benralizumab, a monoclonal antibody targeting the α subunit of the IL-5 receptor. 

The response to biologic treatment in patients with severe asthma should be evaluated at 4–6 months after initiation. If a good response is not achieved in terms of exacerbations, symptom control, lung function, reduced dose of oral corticosteroids and patient satisfaction, then we can consider switching to another biologic, provided that the patient is a suitable candidate [7]. Indeed, several studies have tested the therapeutic switch between two biologics with excellent results, further demonstrating the heterogeneity of asthmatic disease and the complexity of the therapeutic choice [8,9,10]. 

Therefore, based on the above considerations, we decided to evaluate, within a multicentre real-life setting, the potential effectiveness of a therapeutic switch to benralizumab on clinical, functional and laboratory parameters in patients with severe eosinophilic asthma, poorly controlled by previous treatment with mepolizumab. The secondary aim was to identify the clinical factors associated with a good response to benralizumab. 

## 2. Materials and Methods

### 2.1. Study Design

The study was a real-life, retrospective, multicentre Italian study of 25 adult SA patients [11], who had poorly controlled asthma, despite the add-on biological treatment with mepolizumab, and thus switched to benralizumab without a wash-out period. These subjects referred to six Respiratory and Allergology Units of Southern Italy (Foggia, Bari, Catania, Catanzaro, Salerno and Palermo). This study was carried out according to the principles of the Declaration of Helsinki. 

At the time of enrolment, no monoclonal antibodies targeting the IL-5 cascade, other than mepolizumab, were marketed in Italy. At baseline, all the subject enrolled exhibited blood eosinophil counts of at least 150 cells/µL before starting mepolizumab and at least 300 cells/µL during the previous 12 months, and experienced frequent asthma exacerbations and inadequate symptom control, thus needing high dosages of inhaled corticosteroids (ICSs)/long-acting 2-adrenergic agonists (LABAs) combinations, eventually associated with long-acting muscarinic antagonists (LAMAs). Therefore, mepolizumab was prescribed at the licensed dose of 100 mg and administered every 28 days. After a treatment period of at least 12 months, patients were shifted to benralizumab, with a regimen schedule consisting of a single subcutaneous injection of 30 mg every 4 weeks for the first three times, followed by the same dosage given every 8 weeks thereafter, because of their suboptimal response to anti-IL5 therapy. Mepolizumab failure, defined as a partial or total lack of clinical remission (i.e., frequent severe exacerbations and/or poorly controlled symptoms and/or higher OCS daily use in patients with a poor or moderate response in the global evaluation of treatment effectiveness scale), after at least 12 months of treatment [12]. 

Patients were excluded if they had a poor controlled asthma due to inadequate or inappropriate treatment, poor compliance to inhaled therapy or persistent poorly controlled comorbidities, or if they were diagnosed with asthma/chronic obstructive pulmonary disease overlap, or also if any other severe disease was likely to interfere with the conduct of the study.

Data at baseline (i.e., before any biological treatment), as well as after at least 12 months of treatment with mepolizumab, were collected in a retrospective manner. Data after at least 12 months of benralizumab therapy were collected at each patient’s last visit. 

The numbers of asthma exacerbations and hospitalizations, the use of short-acting 2-agonist (SABA) rescue medications, as well as prednisone intake, asthma control test (ACT) score, forced expiratory volume in the first second (FEV1), fractional exhaled nitric oxide (FeNO) levels and blood eosinophil counts were recorded at baseline, after 6 and 12 months of mepolizumab therapy (pre-shift), and at 6 and 12 months after the first injection of benralizumab. Minimal clinically important differences (MCID) were calculated to assess the patients’ response to the two biological treatments according to the criteria suggested by Bonini et al. [13]. Moreover, the sino-nasal outcome test-22 (SNOT-22) questionnaire was administered to patients with chronic rhinosinusitis with nasal polyps (CRSwNP).

### 2.2. Statistical Analysis

All numerical data are expressed as the mean ± standard deviations, and categorical data as percentages. The sample distribution was assessed by means of the Shapiro–Wilk normality test. Subsequently, the data were compared over time by paired sample T-test and Mann–Whitney U-test according to statistical appropriateness. The whole sample of enrolled patients was subsequently divided into two subgroups to assess the efficacy of the biological drugs according to the presence of nasal polyposis: patients reporting a history of nasal polyposis were classified as Group A; conversely, patients without nasal polyposis were Group B. The characteristics at baseline of the two subgroups were compared with unpaired sample tests, while changes after 12 months of treatment with each biologic were assessed with paired sample tests.

All data were analysed using GraphPad (v. 8, GraphPad Software Inc., La Jolla, CA, USA) and a *p* < 0.05 was considered statistically significant.

## 3. Results

### 3.1. Study Population at Baseline

Table 1 summarises the characteristics of all 25 patients (65% female), aged 56.76 ± 11.97 years, enrolled in the study. At baseline, 57% reported history of atopy and 48% of CRSwNP. All participants were treated with ICS/LABA and 87% also with LAMA. Of the entire population, 70% was treated with OCSs and reported an intake of 2.63 ± 1.71 puffs/daily of SABA in the last month used as needed rescue medications. In addition, the participants showed poor control of asthma symptoms also at the asthma control test (ACT score = 11.91 ± 3.50 points).

The subjects performed PFTs that showed an FEV1 of 1.57 ± 0.60 L (56.93 ± 18.07% of pred.) and FVC of 2.69 ± 1.07 L (78.57 ± 22.27% of pred.); laboratory test showed a FeNO values of 73.64 ± 45.11 ppb and total eosinophils count of 660.18 ± 675.75 cell/mcL. Overall, the population studied reported 5.91 ± 3.73 exacerbations/year with 0.26 ± 0.45 hospitalisations at baseline.

### 3.2. Effectiveness of Mepolizumab and Benralizumab at 6 and 12 Months

When compared to the baseline, during biological therapy with mepolizumab (pre-switch period), the number of exacerbations recorded in the previous year decreased from 5.91 ± 3.73 to 1.18 ± 1.62 (*p* < 0.0001) at 6 months and 1.25 ± 1.29 (*p* < 0.0001) at 12 months, the prednisone median dosage decreased from 17.16 ± 9.52 to 8.75 ± 8.66 (*p* < 0.0001) at 12 months, the total eosinophils count decreased from 660.18 ± 675.75 to 116.22 ± 87.42 (*p* < 0.0001) at 6 months and 92.71 ± 46.32 (*p* < 0.0001) at 12 months, the ACT score increased from 11.91 ± 3.50 to 16.77 ± 3.48 (*p* < 0.0001) at 6 months and 18.44 ± 3.39 (*p* < 0.0001) at 12 months, but the patients did not achieve good symptoms control (Table 2). After at least one year of add-on therapy with benralizumab (post-switch period), the number of exacerbations recorded in the previous year decreased to 0.15 ± 0.37 (*p* < 0.0001) at 12 months, the mean number of daily SABA inhalations, OCS intake and the prednisone median dosage significantly decreased from the baseline at 6 months and 12 months, the ACT score increased to 20.24 ± 3.38 (*p* < 0.0001) at 6 months and 20.50 ± 3.45 (*p* < 0.0001) at 12 months, the total eosinophil count decreased to 8.00 ± 30.98 (*p* < 0.0001) at 6 months and 5.23 ± 18.86 (*p* < 0.0001) at 12 months, the FEV1 and FEF_25–75_ significantly increased at 6 month and 12 months compared with baseline. No significant differences were detected in the use of LAMA and in the ICS dosage (Table 2). 

**Figure 1:**
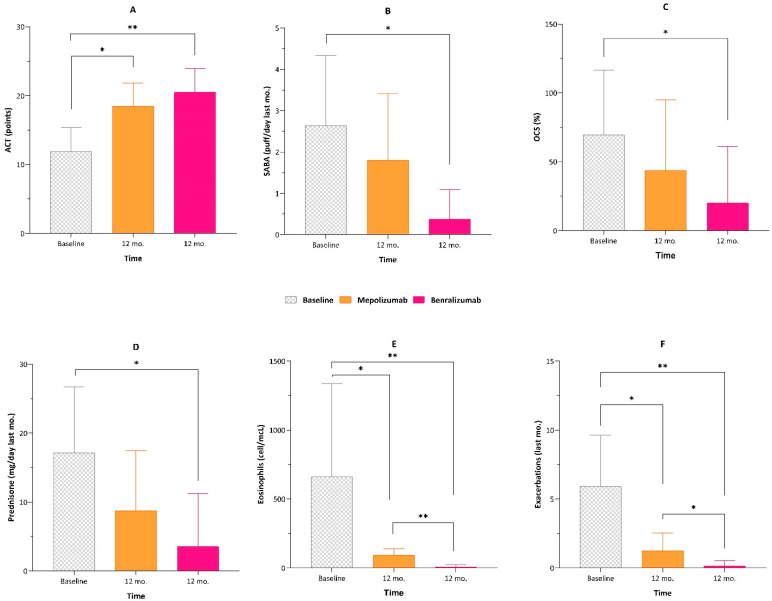
Comparison between the baseline and 12 months of treatment with both mepolizumab and benralizumab in relation to (**A**) the asthma control test (ACT), (**B**) SABA use, (**C**) OCS use, (**D**) prednisone dose intake, (**E**) eosinophilic count and (**F**) number of exacerbations. * *p* < 0.05; ** *p* < 0.01.

The two biologics were compared at 12 months from the initiation of each treatment (Figure 1). 

**Figure 2:**
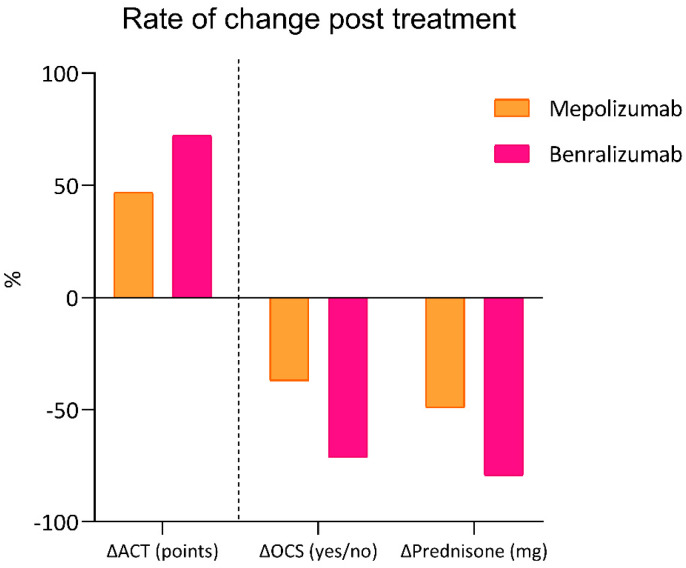
Changes in symptom control (asthma control test) and oral corticosteroid intake (OCS and prednisone dosage) after 12−month treatment with mepolizumab and benralizumab.

At 6 months of treatment, the ACT score with benralizumab was significantly higher than the ACT score with mepolizumab (20.24 ± 3.38 vs. 16.77 ± 3.48, *p* < 0.0001), while at 12 months there was no significant difference between the two values; the mean number of daily SABA inhalations was significantly lower after 6 months and 12 months of treatment with benralizumab than that after treatment with mepolizumab; OCS intake and the prednisone median dosage at 6 months of treatment with benralizumab were significantly lower than those with mepolizumab, while at 12 months there was no significant difference between the two treatments; the total eosinophils count and number of exacerbations recorded in the previous year were significantly lower after 6 months and 12 months of treatment with benralizumab than those after treatment with mepolizumab. However, when analysing the percentage gain in terms of symptom control and OCS tapering at 12 months for both biologics, an increase in ACT of ~50% for mepolizumab and ~70% for benralizumab was observed, while the reductions in OCS and mean prednisone dose were ~40% and ~70% for mepolizumab, and ~50% and ~80% for benralizumab, respectively (Figure 2).

**Figure 3:**
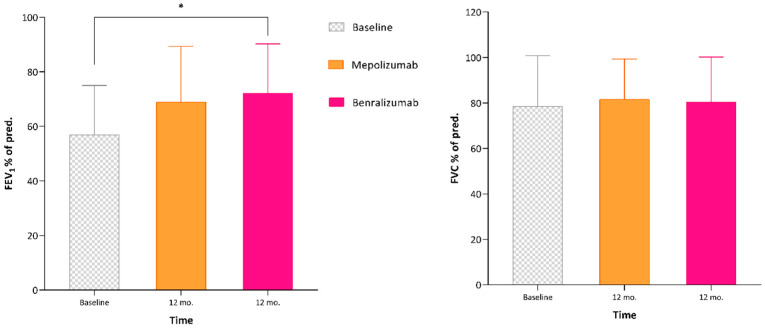
Variations in respiratory function after 12 months of both treatments (mepolizumab and benralizumab, respectively). * *p* < 0.05.

The MCIDs at 12 months of treatment were 73.3% and 88.2% for mepolizumab and benralizumab, respectively. The FEV1 (Figure 3) after 6 months of treatment with benralizumab was significantly higher than that after treatment with mepolizumab, while no differences were detected at 12 months, much less in the FVC and FEF_25–75_.

### 3.3. Effectiveness of Mepolizumab and Benralizumab in Patients with SEA and CRSwNP

We divided the population in two groups according to the history of CRSwNP: Group A (CRSwNP was reported) and Group B (no CRSwNP) (Table 3).

In group A, compared to the baseline, 12-month mepolizumab and benralizumab treatment significantly decreased the number of recurrences of CRSwNP (mepolizumab from 1.44 ± 0.88 to 0.17 ± 0.41, *p* < 0.05; benralizumab from 1.44 ± 0.88 to 0.29 ± 0.49, *p* < 0.05), and increased the ACT score (mepolizumab from 12 ± 4.4 to 18.25 ± 3.54, *p* < 0.05; benralizumab from 12 ± 4.4 to 21.27 ± 3.2, *p* < 0.05). No differences were detected in the LAMA use, the ICS dosage and SNOT-22 score.

At 12 months, the two biologics of treatment decreased the total eosinophil count (mepolizumab from 513.56 ± 291.55 to 95 ± 33.04, *p* < 0.05; benralizumab from 513.56 ± 291.55 to 7.56 ± 22.67, *p* < 0.05) and the number of exacerbations recorded in the previous year (mepolizumab from 6.36 ± 4.72 to 1 ± 1.2, *p* < 0.05; benralizumab from 6.36 ± 4.72 to 0.09 ± 0.3, *p* < 0.05), compared with the baseline. The values were consistently lower with benralizumab than mepolizumab (Table 4).

## 4. Discussion

Our real-life study confirmed the effectiveness of treatment with both anti-IL5 biologics (mepolizumab and benralizumab) in patients with severe eosinophilic asthma. 

However, some patients with severe asthma, notwithstanding the biological therapy prescribed as the first line, still present poorly controlled asthma symptoms and experience a suboptimal clinical response. This is a common issue for patients with severe asthma, since careful clinical phenotyping and endotyping, and consequently choosing a targeted biological treatment are always challenging due to the complex mechanisms underlying the disease. This is especially true for patients who are eligible for either mepolizumab or benralizumab that share the same target, IL-5, which regulates the proliferation, maturation, activation, recruitment and survival of eosinophils that release inflammatory mediators and worsen the clinical condition of asthmatic individuals [14]. These two biologics target IL5 with different mechanisms: mepolizumab binds directly to the IL-5, whereas benralizumab performs its function by binding to the IL-5 receptor α (IL-5Rα) [5]. Indeed, despite several clinical characteristics and biomarkers being described to guide clinicians in selecting the most tailored biological treatment based on super-responder criteria, in some patients, there is still the need for switching biological therapies.

Our multicentre study involved 25 subjects with SEA treated as the first line with mepolizumab at least for one year and then switched to benralizumab because they achieved suboptimal symptom control. 

We chose such a relatively long period of initial treatment because a clinical evaluation restricted to only 4 months, as suggested by the ERS/ATS guidelines [11], carries the risk of considering as a non-responder a potential late responder to a biologic [15]. Furthermore, our patients showed a satisfactory response with mepolizumab in terms of inflammation and the number of exacerbations, but a suboptimal response in terms of symptoms control.

Our findings suggest that benralizumab significantly decreased the annual number of asthma exacerbations, OCS intake, the SABA daily inhalations, utilized as rescue medication, and the total eosinophilic count, not only in comparison to the baseline, but especially with respect to the effects of mepolizumab. Recent studies demonstrated that benralizumab is an effective pharmacotherapeutic option for add-on biological therapy of severe eosinophilic asthma because its rapid therapeutic action, displayed in Table 4, weeks after the first drug administration [16]. A recent study aimed to assess whether elderly patients with SEA could experience an improved asthma control and lung function when switching directly from mepolizumab to benralizumab. They demonstrated that the switch from mepolizumab to benralizumab caused a near-complete reduction in the eosinophil count, but no difference in the treatment response [17].

With respect to mepolizumab treatment and in addition to the positive impact on the annual asthma exacerbation rate, our study demonstrated that benralizumab also elicited other clinical improvements, especially regarding symptom control. Indeed, our patients were characterized by a poor control of asthma symptoms, expressed by a quite low baseline ACT score, which modestly and non-significantly increased after mepolizumab treatment, whereas benralizumab induced a significant improvement compared to the baseline and also versus the ACT score collected during mepolizumab therapy. Our findings are supported by recent studies that demonstrated a reduction in OCS intake and an adequate asthma control after benralizumab administration, resulting in a better quality of life and this could explain a perception by the patient of a better control of symptoms [18]. 

Studies have shown how mepolizumab is effective in patients with eosinophilia at the baseline. Firstly, blood eosinophils were assessed as a biomarker of response due to the simplicity of collecting the sample. However, these are highly variable, so it is desirable to research eosinophils in sputum, but few laboratories can analyse sputum. Thus, peripheral eosinophil counts have a limited role in monitoring response to treatment. Another fact to consider is that several studies have evaluated a reduction in exacerbations of about 50% despite zero eosinophils during treatment with mepolizumab. These data support the hypothesis that eosinophils should not be the only biomarker in T2-high inflammation, but other proinflammatory cytokines should be considered. Benralizumab on the other hand, in addition to its action on peripheral eosinophils and sputum, seems to have a role in reducing basophils in the blood [19]. 

Regarding lung function, our patients were characterized by a relevant airflow limitation at the baseline (mean pre-bronchodilator FEV1: 57% pred). The initial treatment with mepolizumab induced a non-significant FEV1 change, which was overwhelmed by the subsequent therapy with benralizumab, responsible for a significant FEV1 increase after 6 months to more than 500 mL with respect to baseline, and to more than 200 mL when compared to the end of mepolizumab treatment.

These results are in line with recent similar data emerging from both RCT and real-world investigations, showing that mepolizumab is effective in improving the lung function [20,21]. A recent post hoc analysis of an Italian, multicentre, observational, retrospective study, ANANKE, demonstrated that patients who switched to benralizumab because of suboptimal control with a previous biologic therapy (omalizumab or mepolizumab) were more likely to be atopic and showed ACT improvement (≥7 points gained in 48 weeks) and lung function (≥300 mL of FEV1 improvement in 48 weeks) were observed after benralizumab introduction [22].

Finally, we analysed the subgroup of patients with SEA and CRSwNP, and although no differences were detected within the SNOT-22 score and number of recurrences, we confirmed that benralizumab treatment resulted in a marked improvement in asthma control, suppressed blood eosinophil levels and reduction in the number of exacerbations. Similar findings are reported in a recent case report of Mansur AH [23]. Another interesting finding observed is the reduction in FeNO; even literature data are still unclear on this. In fact, a recent systematic review and meta-analysis reported low evidence in this regard [24]. Just as we have noticed, a functional improvement of the small airways was confirmed in some previous studies [25].

To date, no clinical differences between responders and non-responders to mepolizumab have been identified that predict the subsequent response to benralizumab [24].

The strengths of our study include the multicentre design, with patients from eight severe asthma centres across Italy. The main limitations of our study are related to the small number of patients and to its retrospective design. Moreover, no washout period between the treatments was registered; therefore, we cannot really establish how much previous therapy with mepolizumab contributed to determining the positive result of the subsequent benralizumab therapy, if there is a “combined” effects due to the sequential use of the two treatments or if the different mechanism of action of mepolizumab and benralizumab on IL-5 can explain the different results obtained. However, the year-long positive effect certainly confirms the efficacy of treatment with benralizumab. In addition, benralizumab was more recently released and approved for reimbursement for severe asthma treatment compared with mepolizumab; therefore, some patients were initially prescribed with mepolizumab because of the only anti-IL-5 available at the time of patient presentation, or the patients may not have been extensively phenotyped for biologics at the beginning of treatment as they might be at present, following advances in medical knowledge in terms of super responders to biological treatment [26]. 

In conclusion, the patients diagnosed with SEA who experienced a partial response with mepolizumab could benefit from switching to benralizumab, and even more those who have nasal polyposis. This suggests that this approach can reduce the number of exacerbations and corticosteroid cycles, and improve the control of asthma, with economic benefits. This strategy is favoured by the possibility of switching patients safely from one biologic to another which shares the same target, but acts through a different mechanism, without a wash-out period. However, further studies are needed to test this switch in larger population samples, perhaps even using oscillometry for an in-depth study of the small airways.

## Figures and Tables

**Table 1:** General characteristics of the population at baseline.

Variable	Total (*N* = 25)
Demographic	
Age (years)	56.76 ± 11.97
Sex (% female)	64%
BMI (kg/m^2^)	26.23 ± 5.74
Smoking (%)	39%
Pack/years (no.)	9.77 ± 6.29
Medical history	
Asthma (years of onset)	34.48 ± 17.60
Asthma, familiar history (%)	48%
Menopause (%)	46%
Atopy (%)	57%
ASA sensitivity (%)	9%
EGPA (%)	9%
Hives (%)	9%
Dermatitis (%)	9%
GERD (%)	26%
OSAS (%)	13%
Osteoporosis (%)	22%
Anxiety/Depression (%)	47%
Bronchiectasis (%)	35%
CRSwNP	
CRSwNP (%)	48%
Lund–Mackay score (points)	8.00 ± 5.94
SNOT-22 (points)	51.33 ± 17.18
VAS (points)	8.67 ± 1.15
Nasal Polyp Score (points)	3.00 ± 2.65
Recurrence (n°)	1.44 ± 0.88
Current therapy	
ICS/LABA (%)	100%
LAMA (%)	87%
SABA (puff/day last mo.)	2.63 ± 1.71
LTRA (%)	57%
Azithromycin (%)	17%
OCS (%)	70%
Prednisone (mg/day last mo.)	17.16 ± 9.52
Immunotherapy (%)	4%
Methotrexate (%)	20%
Questionnaires	
ACT (points)	11.91 ± 3.50
ACQ (points)	3.22 ± 1.58
AQLQ (points)	5.52 ± 0.69
TAI (points)	49.67 ± 0.58
Pulmonary Function	
FEV_1_ (% of pred.)	56.93 ± 18.07
FEV_1_ pre (L)	1.57 ± 0.60
FVC (% of pred.)	78.57 ± 22.27
FVC pre (L)	2.69 ± 1.07
FEV_1_/FVC%	60.97 ± 14.44
FEF_25–75_ (% of pred.)	26.16 ± 17.88
Biomarkers	
FeNO (ppb)	73.6 ± 45.1
Eosinophils (cell/mcL)	660.2 ± 675.7
Basophils (cell/mcL)	51.8 ± 24.5
Neutrophils (cell/mcL)	4815.1 ± 1415.2
Total IgE (IU/mL)	446.0 ± 653.49
Exacerbations	
Exacerbations (no.)	5.91 ± 3.73
A&E (no. admission)	0.91 ± 1.23
Hospitalisation (no. admission)	0.26 ± 0.45

Data are expressed as the mean ± standard deviation, number (no.) and percentage. Abbreviations: A&E = accident and emergency department; ACT = asthma control test; ACQ = asthma control questionnaire; AQLQ = asthma quality of life questionnaire; CRSwNP = chronic rhinosinusitis with nasal polyps; EGPA = eosinophilic granulomatosis with polyangiitis; FEF_25–75_ = forced expiratory flow at 25 and 75% of the pulmonary volume; FeNO = fractional exhaled nitric oxide; FEV_1_ = forced expiratory volume in the 1st second; FVC = forced vital capacity; GERD = gastroesophageal reflux disease; ICS/LABA = inhaled corticosteroid/long-acting β2-agonist (LABA); Ig IV = intravenous immunoglobulin; LAMA = long-acting muscarinic antagonists; LTRA = leukotriene receptor antagonists; OCS = oral corticosteroid; OSAS = obstructive sleep apnoea syndrome; RICU = respiratory intensive care unit; SABA = short-acting b2 agonist; SNOT-22 = sinonasal outcome test 22; TAI = test of adherence to inhalers; VAS = visual analogue scale.

**Table 2:** Effectiveness of mepolizumab and benralizumab at 6 and 12 months.

Variable	Population (*N* = 25)	Mepolizumab		Benralizumab	
Timeline (mo.)	Baseline	6 mo.	12 mo.	6 mo.	12 mo.
Current therapy					
ICS/LABA (%)	100%	100%	100%	100%	100%
LAMA (%)	87%	73%	69%	70%	70%
SABA (puff/day last mo.)	2.63 ± 1.71	1.50 ± 1.76 ^e^	1.80 ± 1.61	0.19 ± 0.40 ^c^*^,e^	0.38 ± 0.72 ^d^*^,f^
LTRA (%)	57%	55%	69%	65%	70%
Azithromycin (%)	17%	9%	19%	10%	5%
Theophylline (%)	13%	9%	13%	10%	10%
OCS (%)	70%	59% ^e^	44%	15% ^c^*^,e^	20% ^d^*
Prednisone (mg/day last mo.)	17.16 ± 9.52	12.75 ± 9.62 ^e^	8.75 ± 8.66 ^b^	2.95 ± 5.14 ^c^*^,e^	3.54 ± 7.72 ^d^*
Immunotherapy (%)	4%	0%	0%	0%	0%
Questionnaires					
ACT (points)	11.91 ± 3.50	16.77 ± 3.48 ^a^*^,e^	18.44 ± 3.39 ^b^*	20.24 ± 3.38 ^c^*^,e^	20.50 ± 3.45 ^d^*
ACQ (points)	3.22 ± 1.58	2.54 ± 0.99 ^e^	1.36 ± 1.15 ^b^	0.90 ± 0.97 ^c,e^	1.92 ± 2.15
AQLQ (points)	5.52 ± 0.69	4.50 ± 1.78	5.86 ± 0.77	7.00 ± 1.14	5.95 ± 0.89
TAI (points)	49.67 ± 0.58	49.67 ± 0.58	49.00 ± 1.32	50.00 ± 0.00	52.67 ± 2.31
CRSwNP					
CRSwNP (%)	48%	69%	73%	75%	75%
SNOT-22 (points)	51.33 ± 17.18	31.00 ± 1.41	45.00 ± 8.28	20.67 ± 9.29 ^c^	38.80 ± 18.25
Recurrence (no.)	1.44 ± 0.88	0.50 ± 1.00	0.17 ± 0.41 ^b^	0.20 ± 0.45	0.25 ± 0.46 ^d^
Pulmonary Function Test					
FEV_1_ (% of pred.)	56.93 ± 18.07	58.36 ± 19.56 ^e^	69.00 ± 20.32	73.20 ± 16.52 ^c,e^	72.18 ± 18.06 ^d^
FEV_1_ pre (L)	1.57 ± 0.60	1.59 ± 0.64 ^e^	1.87 ± 0.70	2.13 ± 0.70 ^c,e^	2.14 ± 0.71 ^d^
FVC (% of pred.)	78.57 ± 22.27	76.33 ± 16.95	81.57 ± 17.74	81.27 ± 25.52	80.35 ± 19.88
FVC pre (L)	2.69 ± 1.07	2.53 ± 0.77	2.80 ± 0.87	10.40 ± 28.12	2.96 ± 0.96
FEV_1_/FVC%	60.97 ± 14.44	63.88 ± 17.75	67.35 ± 12.79	69.96 ± 15.24	69.66 ± 24.30
FEF_25–75_ (% of pred.)	26.16 ± 17.88	30.33 ± 19.87	34.18 ± 20.31	43.13 ± 23.94 ^c^	48.00 ± 23.94 ^d^
Biomarkers					
FeNO (ppb)	73.6 ± 45.1	49.24 ± 38.9	51.90 ± 46.59	34.45 ± 27.81	30.17 ± 16.51
Eosinophils (cell/mcL)	660.2 ± 675.7	116.22 ± 87.42 ^a,e^*	92.71 ± 46.32 ^b^	8.0 ± 30.98 ^c^*^,e^*	5.23 ± 18.86 ^d,f^*
Basophils (cell/mcL)	51.8 ± 24.5	11.75 ± 9.3 ^a^*	18.4 ± 12.36 ^b^	6.0 ± 6.35 ^c^*	5.63 ± 10.28 ^d^*
Neutrophils (cell/mcL)	4815.2 ± 1415.3	4150.3 ± 1031.36	4288.33 ± 1225.71	4520.0 ± 800.17	5112.0 ± 1500.24
Exacerbations					
Exacerbation (no.)	5.91 ± 3.73	1.18 ± 1.62 ^a^*^,e^	1.25 ± 1.29 ^b^*	0 ^c^*^,e^	0.15 ± 0.37 ^d^*^,f^*
A&E (no. admission)	0.91 ± 1.23	0.17 ± 0.51 ^a^	0.07 ± 0.26 ^b^	0 ^c^	0 ^d^
Hospitalisation (no. admission)	0.26 ± 0.45	0 ^a^	0.07 ± 0.26	0 ^c^	0 ^d^

Data are expressed as the mean ± standard deviation, number (no.) and percentage. Significant differences between the groups: ^a^ *p* < 0.05 between the baseline and mepolizumab (6 mo.); ^a^* *p* < 0.001 between the baseline and mepolizumab (6 mo.) ^b^ *p* < 0.05 between the baseline and mepolizumab (12 mo.); ^b^* *p* < 0.001 between the baseline and mepolizumab (12 mo.) ^c^ *p* < 0.05 between the baseline and benralizumab (6 mo.); ^c^* *p* < 0.001 between the baseline and benralizumab (6 mo.) ^d^ *p* < 0.05 between the baseline and benralizumab (12 mo.); ^d^* *p* < 0.001 between the baseline and benralizumab (12 mo.) ^e^ *p* < 0.05 between mepolizumab and benralizumab at 6 mo.; ^e^* *p* < 0.001 between mepolizumab and benralizumab at 6 mo. ^f^ *p* < 0.05 between mepolizumab and benralizumab at 12 mo.; ^f^* *p* < 0.001 between mepolizumab and benralizumab at 12 mo. Abbreviations. A&E = accident and emergency department; ACT = asthma control test; ACQ = asthma control questionnaire; AQLQ = asthma quality of life questionnaire; CRSwNP = chronic rhinosinusitis with nasal polyps; FEF_25–75_ = forced expiratory flow at 25 and 75% of the pulmonary volume; FeNO = fractional exhaled nitric oxide; FEV_1_ = forced expiratory volume in the 1st second; FVC = forced vital capacity; ICS/LABA = inhaled corticosteroid/long-acting β2-agonist (LABA); LAMA = long-acting muscarinic antagonists; LTRA = leukotriene receptor antagonists; OCS = oral corticosteroid; RICU = respiratory intensive care unit; SABA = short-acting b2 agonist; SNOT-22 = sinonasal outcome test 22; TAI = test of adherence to inhalers.

**Table 3:** Characteristics of patients with and without CRSwNP at baseline.

Variable	Group A (CRSwNP)	Group B (no CRSwNP)	*p*
	*N* = 12	*N* = 13	
Current therapy			
ICS/LABA (%)	100%	100%	—
LAMA (%)	100%	75%	0.082
SABA (puff/day last mo.)	2.78 ± 1.92	2.5 ± 1.58	0.708
LTRA (%)	55%	58%	0.863
Azithromycin (%)	27%	8%	0.251
OCS (%)	82%	58%	0.240
Prednisone (mg/day last mo.)	16.5 ± 11.07	18.11 ± 7.51	0.685
Immunotherapy (%)	0%	8%	0.350
Questionnaires			
ACT (points)	12 ± 4.4	11.83 ± 2.76	0.914
ACQ (points)	2.48 ± 1.78	4.2 ± 0.4	0.004
Pulmonary Function Test			
FEV_1_ (% of pred.)	64.09 ± 21.27	50.38 ± 11.98	0.067
FEV_1_ pre (L)	1.93 ± 0.6	1.24 ± 0.38	0.003
FVC (% of pred.)	88 ± 25.51	69.92 ± 15.15	0.049
FVC pre (L)	3.37 ± 1.15	2.07 ± 0.45	0.002
FEV_1_/FVC%	62.02 ± 16.02	60 ± 13.46	0.746
FEF_25–75_ (% of pred.)	33.25 ± 21.57	21 ± 13.44	0.144
Biomarkers			
FeNO (ppb)	73.38 ± 46.84	74.33 ± 49.92	0.963
Eosinophils (cell/mcL)	513.56 ± 291.55	794.58 ± 891.52	0.331
Basophils (cell/mcL)	71.6 ± 21.74	35.33 ± 10.39	<0.001
Neutrophils (cell/mcL)	4762.0 ± 1009.1	4868.33 ± 1838.88	0.867
Total IgE (IU/mL)	584.0 ± 878.58	308.01 ± 304.49	0.317
Exacerbations			
Exacerbation (no.)	6.36 ± 4.72	5.5 ± 2.68	0.591
A&E (no. admission)	1.18 ± 1.47	0.64 ± 0.92	0.295
Hospitalisation (no. admission)	0.22 ± 0.44	0.3 ± 0.48	0.692

Data are expressed as the mean ± standard deviation, number (no.) and percentage. Abbreviations. A&E = accident and emergency department; ACT = asthma control test; ACQ = asthma control questionnaire; AQLQ = asthma quality of life questionnaire; CRSwNP = chronic rhinosinusitis with nasal polyps; FEF_25–75_ = forced expiratory flow at 25 and 75% of the pulmonary volume; FeNO = fractional exhaled nitric oxide; FEV_1_ = forced expiratory volume in the 1st second; FVC = forced vital capacity; ICS/LABA = inhaled corticosteroid/long-acting β2-agonist (LABA); LAMA = long-acting muscarinic antagonists; LTRA = leukotriene receptor antagonists; OCS = oral corticosteroid; RICU = respiratory intensive care unit; SABA = short-acting b2 agonist; SNOT-22 = sinonasal outcome test 22; TAI = test of adherence to inhalers.

**Table 4:** Effectiveness of mepolizumab and benralizumab at 12 months in patients with SEA and CRSwNP.

Variable	Baseline	Mepolizumab	Benralizumab
Current therapy			
ICS/LABA (%)	100%	100%	100%
LAMA (%)	100%	75%	73%
SABA (puff/day last mo.)	2.78 ± 1.92	1.5 ± 1.69	0.22 ± 0.67 ^β^
LTRA (%)	55%	75%	73%
Azithromycin (%)	27%	13%	9%
OCS (%)	82%	50%	27% ^β^
Prednisone (mg/day last mo.)	16.5 ± 11.07	7.5 ± 5.92	4.17 ± 8.84 ^β^
Questionnaires			
ACT (points)	12 ± 4.4	18.25 ± 3.54 ^α^	21.27 ± 3.2 ^β^
ACQ (points)	2.48 ± 1.78	1.02 ± 1.06	2.17 ± 2.57
AQLQ (points)	5.52 ± 0.69	5.86 ± 0.77	6.21 ± 0.88
CRSwNP			
SNOT-22 (points)	51.33 ± 17.18	45 ± 8.28	38.8 ± 18.25
Recurrence (no.)	1.44 ± 0.88	0.17 ± 0.41 ^α^	0.29 ± 0.49 ^β^
Pulmonary Function			
FEV_1_ (% of pred.)	64.09 ± 21.27	76.25 ± 22.94	78.36 ± 15.36
FEV_1_ pre (L)	1.93 ± 0.6	2.18 ± 0.69	2.39 ± 0.63
FVC (% of pred.)	88 ± 25.51	90.13 ± 16.79	89.45 ± 13.53
FVC pre (L)	3.37 ± 1.15	3.3 ± 0.68	3.28 ± 0.91
FEV_1_/FVC%	62.02 ± 16.02	65.61 ± 14.16	67.66 ± 29.22
FEF_25–75_ (% of pred.)	33.25 ± 21.57	33.6 ± 27	53.36 ± 24.44
Biomarkers			
FeNO (ppb)	73.38 ± 46.84	61.85 ± 56.47	24.95 ± 10.9
Eosinophils (cell/mcL)	513.56 ± 291.55	95.0 ± 33.04 ^α,γ^	7.56 ± 22.67 ^β,γ^
Exacerbations			
Exacerbation (no.)	6.36 ± 4.72	1 ± 1.2 ^α,γ^	0.09 ± 0.3 ^β,γ^
A&E (no. admission)	1.18 ± 1.47	0.13 ± 0.35	0 ^β^
Hospitalisation (no. admission)	0.22 ± 0.44	0.13 ± 0.35	0

Data are expressed as the mean ± standard deviation, number (no.) and percentage. Significant differences between the groups: ^α^ A *p*-value < 0.05 was found between the baseline and mepolizumab; ^β^ A *p*-value < 0.001 was found between the baseline and benralizumab for ACT, eosinophils, basophils and no. exacerbations, while a *p*-value < 0.05 for the other comparisons; ^γ^ A *p*-value < 0.001 was found between mepolizumab and benralizumab for eosinophils, while a *p*-value < 0.05 was found for the no. of exacerbations. Abbreviations. A&E = accident and emergency department; ACT = asthma control test; ACQ = asthma control questionnaire; AQLQ = asthma quality of life questionnaire; CRSwNP = chronic rhinosinusitis with nasal polyps; FEF_25–75_ = forced expiratory flow at 25 and 75% of the pulmonary volume; FeNO = fractional exhaled nitric oxide; FEV_1_ = forced expiratory volume in the 1st second; FVC = forced vital capacity; ICS/LABA = inhaled corticosteroid/long-acting β2-agonist (LABA); LAMA = long-acting muscarinic antagonists; LTRA = leukotriene receptor antagonists; OCS = oral corticosteroid; RICU = respiratory intensive care unit; SABA = short-acting b2 agonist; SNOT-22 = sinonasal outcome test 22.

## Data Availability

The data that support the findings of this study are available from the corresponding author upon reasonable request.
